# Glutamic Acid and Poly-γ-glutamic Acid Enhanced the Heat Resistance of Chinese Cabbage (*Brassica rapa* L. ssp. *pekinensis*) by Improving Carotenoid Biosynthesis, Photosynthesis, and ROS Signaling

**DOI:** 10.3390/ijms231911671

**Published:** 2022-10-01

**Authors:** Jin Quan, Weiwei Zheng, Jingru Tan, Zewei Li, Meifang Wu, Seung-Beom Hong, Yanting Zhao, Zhujun Zhu, Yunxiang Zang

**Affiliations:** 1 Key Laboratory of Quality and Safety Control for Subtropical Fruit and Vegetable, Ministry of Agriculture and Rural Affairs, Collaborative Innovation Center for Efficient and Green Production of Agriculture in Mountainous Areas of Zhejiang Province, College of Horticulture Science, Zhejiang A&F University, Hangzhou 311300, China; 2 Department of Biotechnology, University of Houston Clear Lake, Houston, TX 77058-1098, USA; 3 Institute of Vegetables, Zhejiang Academy of Agricultural Sciences, Hangzhou 310021, China

**Keywords:** glutamic acid, poly-γ-glutamic acid, Chinese cabbage, heat stress, photosynthesis, antioxidant enzyme activity, biostimulant

## Abstract

Heat stress is one of the most common agrometeorological risks in crop production in the middle and lower reaches of the Yangtze River in China. This study aimed to investigate whether glutamic acid (Glu) or poly-γ-glutamic acid (γ-PGA) biostimulants can improve the thermotolerance of a cool-season Chinese cabbage (*Brassica rapa* L. ssp. *pekinensis*) crop. Priming with Glu (2.0 mM) or γ-PGA (20 mg·L^−1^) was conducted at the third leaf stage by applying as daily foliar sprays for 5 days before 5 days of heat stress (45 °C in 16-h light/35 °C in 8-h dark). Coupled with morpho-physiological and biochemical analyses, transcriptomes of Glu or γ-PGA-primed Chinese cabbage under heat stress were examined by RNA-seq analysis. The results showed that the thermotolerance conferred by Glu and γ-PGA priming was associated with the increased parameters of vegetative growth, gas exchange, and chlorophyll fluorescence. Compared with the control, the dry weights of plants treated with Glu and γ-PGA increased by 51.52% and 39.39%, respectively. Glu and γ-PGA application also significantly increased the contents of total chlorophyll by 42.21% and 23.12%, and carotenoid by 32.00% and 24.00%, respectively. In addition, Glu- and γ-PGA-primed plants markedly inhibited the levels of malondialdehyde, electrolyte leakage, and super-oxide anion radical, which was accompanied by enhanced activity levels of superoxide dismutase (SOD), catalase (CAT), ascorbate peroxidase (APX), and peroxidase (POD). Enrichment analysis of Kyoto Encyclopedia of Genes and Genomes (KEGG) categories within the differentially expressed genes (DEGs) functional clusters of RNA-seq data indicated that the expression levels of the genes for DNA replication, DNA repair system, linoleic acid metabolism, cysteine and methionine metabolism, glutathione metabolism, purine and pyrimidine metabolism, carotenoid biosynthesis, and plant–pathogen interaction were commonly up-regulated by both Glu and γ-PGA priming. Glu treatment enhanced the expression levels of the genes involved in aliphatic glucosinolate and 2-oxocarboxylic acid, while γ-PGA treatment activated carotenoid cleavage reaction to synthesize abscisic acid. Taken together, both Glu and γ-PGA have great potential for the preadaptation of Chinese cabbage seedlings to heat stress, with Glu being more effective than γ-PGA.

## 1. Introduction

Heat stress is one of the most hazardous abiotic stresses that severely affect plant growth and development [1,2]. At present, the average global warming is about 2 °C above pre-industrial levels [3,4]. It is anticipated that steadily rising temperatures as a result of global climate change will have a negative impact on agricultural productivity. Heat stress inevitably leads to accumulation of reactive oxygen species (ROS) beyond a tolerant level in plant cells, causing progressive oxidative damage to cell membranes, cellular proteins, nucleic acids and enzymes, weakening photosynthetic activity, and ultimately resulting in the loss of yield and quality or crop death [2,5]. Plants have evolved a series of protective enzymatic and non-enzymatic ROS scavenging systems to sustain redox homeostasis between ROS generation and the neutralization of excess ROS, as well as redox signaling as components of maintenance of a normal physiological steady state [6,7]. The enzymatic detoxification mechanism includes production of antioxidant enzymes, such as superoxide dismutase, peroxidase, catalase, ascorbate peroxidase, and glutathione peroxidase [7]. The non-enzymatic detoxification mechanism includes antioxidant metabolites such as glutathione, phenolics, flavonoids, and carotenoids [8]. Nevertheless, plant defense mechanisms are usually not enough to provide adequate protection from high-temperature stresses. Therefore, it is necessary to implement new agronomic strategies to improve the thermotolerance capacity of plants.

As alternative conventional strategies, biostimulants have received considerable attention since they are eco-friendly substances or microorganisms that can effectively protect plants from a variety of biotic/abiotic stresses [9]. They are known to have beneficial impacts on many physiological and biochemical processes of plants, such as photosynthesis, nutrients uptake, stress tolerance via enhanced antioxidant defense system and osmoregulation, and crop yield and quality [10]. Biostimulant products are often formulated from the chemical hydrolysis of raw materials rich in amino acids, among which glutamic acid (Glu) plays a central role in amino acid metabolism as the precursor of other amino acids, such as aspartic acid, serine, alanine, lysine, and proline [11,12]. It is not only involved in the assimilation of nitrogen and the reactions of amino transferases in plants [11] but also the precursor of 5-aminolevulinic acid for chlorophyll synthesis in developing leaves [13,14]. Besides nutrition, exogenous Glu had a positive influence on plants under abiotic stresses by stimulating the biosynthesis of chlorophyll and proline as an osmoprotectant [15,16]. Several studies have pointed out the positive effects of Glu on photosynthesis, carbon/nitrogen balance, Ca^2+^ homeostasis, root architecture, pollen tube growth, and defense signaling under environmental stresses [17,18,19].

Poly-γ-glutamic acid (γ-PGA) is an extracellular polymer secreted as a slime layer of certain Bacillus species that is composed of D- and L-glutamic acid monomers connected by amide linkages between α-amino and γ-carboxyl groups [20]. As another plant biostimulant, γ-PGA is promising in agricultural application fields due to its features of cation chelating, hygoscopicity, water-solubility, non-toxicity, and biodegradability [21]. Several studies have shown that γ-PGA could improve the tolerance of plants to salt, cold, and drought [22,23]. Gene expression analysis suggested that stress tolerance promoted by exogenous γ-PGA was mediated by the enhanced nitrogen assimilation and biosynthesis of brassinosteroids, jasmonic acid, and abscisic acid [23,24,25]. However, research on the effect of exogenous Glu and γ-PGA on thermotolerance capacity of plants is currently limited.

Chinese cabbage (*Brassica rapa* L. ssp. *pekinensis*) is among the major globally produced leafy vegetable crops, and its production and quality are mostly affected by high-temperature stress as a cool-weather plant [26,27]. It is quite tolerant of cold temperatures but does not adapt to heat very well, and its efficient growth temperature is about 22 °C. Chinese cabbage seedlings can tolerate temperatures up to 27 °C [28]. However, the seeds are often sown in early August at a high temperature (over 30 °C) in the Yangtze River area in China [29]. Therefore, improving the heat stress tolerance has always been a matter of significant interest to Chinese cabbage cultivators. To date, there are still very limited studies on the use of biostimulants in Chinese cabbage to improve the thermotolerance capacity of Chinese cabbage.

The objective of the present study was to assess the capacity of Glu and γ-PGA to confer thermotolerance to Chinese cabbage seedlings and reveal its associated changes at morpho-physiological, biochemical, and transcript levels. To this end, biomass, photosynthetic pigment, gas exchange parameters, chlorophyll fluorescence parameters, electrolyte leakage (EL), proline content, malondialdehyde (MDA) content, and antioxidant enzyme activity were measured. In addition, changes in gene expression profiles were examined using RNA sequencing (RNA-seq) to understand the underlying molecular mechanisms of heat tolerance conferred by exogenous Glu or γ-PGA. Our results would provide a technical basis of agronomic management practices for the utilization of Glu and γ-PGA to effectively mitigate high-temperature stress from Chinese cabbage.

## 2. Results

### 2.1. Effect of Glu and γ-PGA on the Growth of Chinese Cabbage Seedlings under Heat Stress

**Figure 1:**
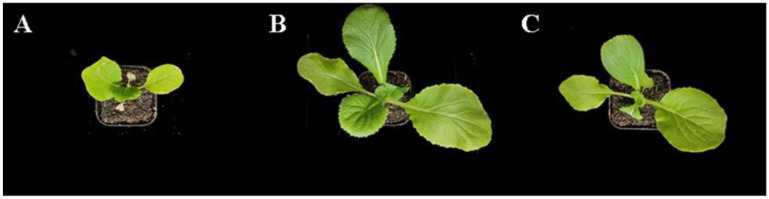
The phenotype of Chinese cabbage treated with water (**A**), Glu (**B**), or γ-PGA (**C**), followed by heat treatment.

Heat-stressed control plants became stunted and wilted, with some leaves being turned yellowish, whereas the plants pretreated with Glu or γ-PGA were healthier and greener, with more expanded leaves, especially with Glu (Figure 1). As compared to the control, Glu and γ-PGA application increased fresh weight by 47.58% and 37.32%, dry weight by 51.52% and 39.39%, plant height by 27.78% and 26.43%, hypocotyl diameter by 13.78% and 9.33%, leaf length by 35.51% and 32.76%, and leaf width by 43.04% and 31.26%, respectively (Table 1). These results showed that Glu and γ-PGA conferred heat tolerance to Chinese cabbage seedlings to promote growth.

### 2.2. Effects of Glu and γ-PGA on MDA, Electrolyte Leakage, and Proline Content under Heat Stress

**Figure 2:**
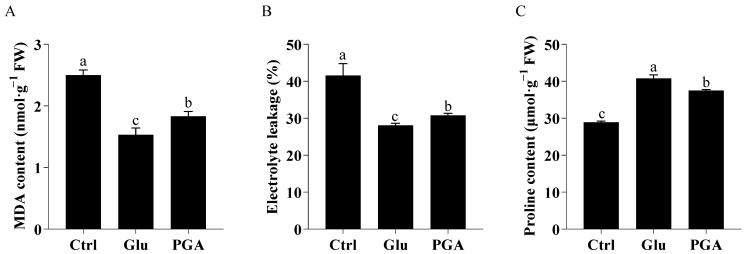
Effect of Glu and γ-PGA on MDA content (**A**), electrolyte leakage (**B**), proline content (**C**) in Chinese cabbage under heat stress. Data are shown as mean ± SD. Different lowercase letters indicate significant difference at *p* < 0.05.

Levels of both MDA and electrolyte leakage reflect the degree of cell membrane injury caused by ROS generated during stresses [30]. Compared to the control, Glu and γ-PGA significantly reduced MDA content by 49.68% and 36.43% and leaf electrolyte leakage by 41.81% and 24.41%, respectively (Figure 2A,B). Proline is known to help plant cells to tolerate stresses by acting as an osmoprotectant and ROS quencher [31]. Glu- and γ-PGA-treated plants increase proline content by 40.93% and 29.68%, respectively (Figure 2C). As a result, Glu treatment appears to be more effective in mitigating heat stress than γ-PGA treatment through a higher capacity of antioxidation and osmoprotection.

### 2.3. Effect of Glu and γ-PGA on Antioxidant Enzyme Activity and Superoxide Radicals under Heat Stress

The levels of antioxidant enzyme activities and superoxide radicals reflect the capacity of plants to detoxify ROS generated during oxidative stress [30]. As shown in Table 2, application of Glu significantly improved the activity of SOD, APX, CAT, and POD by 19.06%, 27.01%, 34.19%, and 45.48%, respectively. Application of γ-PGA significantly increased the activity of SOD, APX, CAT, and POD by 13.30%, 26.12%, 33.21%, and 39.05%, respectively. However, there were no significant differences in the enzyme activities between Glu- and γ-PGA-primed heat-stressed plants. On the other hand, priming with Glu and γ-PGA reduced the O_2_^−^ induced oxidative stress by 49.40% and 29.07% as compared to the control. Thus, γ-PGA conferred a higher capacity of reducing heat-induced oxidative stress to Chinese cabbage plant than Glu.

### 2.4. Effect of Glu and γ-PGA on Photosynthetic Pigment, Gas Exchange Parameters, and Chlorophyll Fluorescence Parameters under Heat Stress

#### 2.4.1. Photosynthetic Pigments

The variations in chlorophyll *a* (chl *a*), chlorophyll *b* (chl *b*), total chlorophyll, and total carotenoids in the Glu- and γ-PGA-primed plants under heat stress are shown in Table 3. Priming with Glu and γ-PGA increased chl *a* by 38.31% and 22.10%, chl *b* by 53.33% and 26.67%, and total chl by 42.21% and 23.12%, respectively. Although the contents of chl *a* are about three times higher than chl *b* in the leaf tissue regardless of the priming chemical treatments, as reported previously [32], the chl *a*/*b* ratio of both Glu- and γ-PGA-primed plants decreased relative to the control. Unlike the spectrophotometrically measured concentrations of total chlorophyll extracts, no significant differences in SPAD values were noted between the plants primed with Glu and γ-PGA, although those SPAD values were significantly higher than that of the control plant. The discrepancy between the spectrophotometrically measured chlorophyll contents and SPAD values in the Glu- and γ-PGA-primed plants could be attributed to the uneven distribution of chlorophyll along the position of the leaf blade used for SPAD meter measurement [33]. As compared to the control, priming with Glu and γ-PGA increased total chlorophyll content by 42.21% and 23.12%, respectively. Glu and γ-PGA priming also increased total carotenoids content by 32.00% and 24.00%, respectively. Thus, the plants primed with Glu and γ-PGA had significantly higher total chlorophyll and carotenoids contents than the non-primed heat-stressed control plant, suggesting that Glu and γ-PGA treatment protected photosynthetic pigments from high-temperature stress since heat-induced decline in pigments was a result of lipid peroxidation of chloroplast and thylakoid membranes [34].

#### 2.4.2. Gas Exchange Parameters

**Figure 3:**
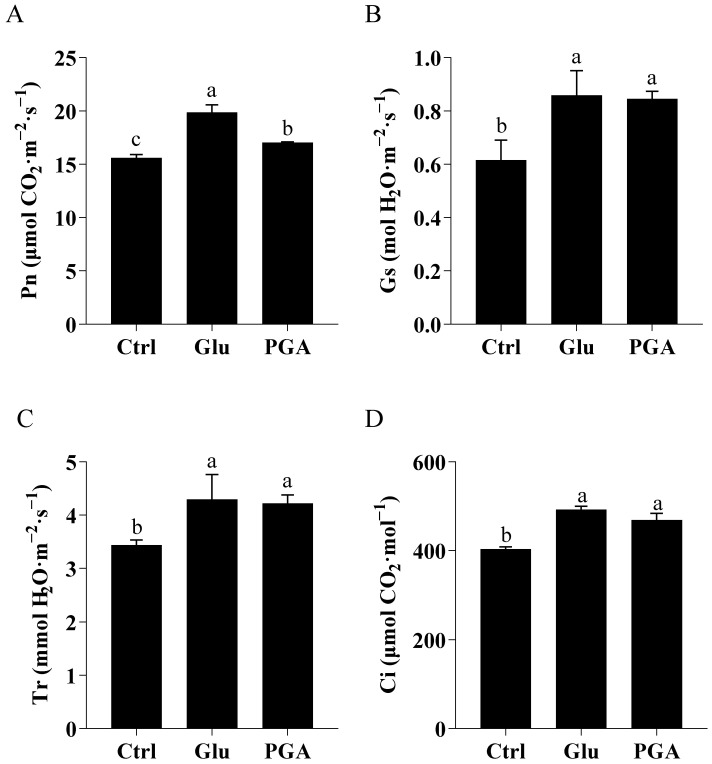
Effect of Glu and γ-PGA on gas exchange parameters in Chinese cabbage under heat stress. Pn (**A**), Gs (**B**), Tr (**C**), Ci (**D**). Data are shown as mean ± SD of three replicates. Different lowercase letters indicate significant differences at *p* < 0.05.

Besides the quantity of photosynthetic pigments, gas exchange is another indicator of photosynthetic capacity that is vulnerable to environmental stresses. Heat markedly influences the leaf water status, Pn, Gs, Tr, and Ci [1]. As shown in Figure 3, Chinese cabbage plants primed with Glu significantly enhanced Pn (29.4%), Gs (30.82%), Tr (19.70%), and Ci (21.98%). Further, γ-PGA treatment also significantly increased Pn (11.76%), Gs (31.90%), Tr (26.87%), and Ci (19.01%). Overall, both Glu and γ-PGA treatments improved the leaf gas-exchange attributes, with higher efficacy of Glu than γ-PGA in plant adaptation to heat stress.

#### 2.4.3. Chlorophyll Fluorescence Parameters

Chlorophyll fluorescence is known to be highly sensitive to external conditions, and, thus, its parameters provide a useful measure of the photosynthetic activity in response to heat stress [35]. In order to investigate the physiological consequences of Glu and γ-PGA application in photosynthesis, photosystem II (PSII) photochemistry parameters of maximum quantum yield of PSII (Fv/Fm and Fv/Fo), actual photochemical efficiency (ФPSII), electron transfer rate (ETR), photochemical quenching (qP), and non-photochemical quenching (NPQ) in the Glu- and γ-PGA-primed heat-stressed plants were measured (Table 4). The Fv/Fo values of Glu and γ-PGA were significantly increased by 29.52% and 18.73%, respectively, compared with the control group. The ratio of Fv/Fm in the control, Glu, and γ-PGA treatments showed lower amplitudes than Fv/Fo, 0.77, 0.80, and 0.79, respectively. As compared to the control, the Glu treatment significantly increased ФPSII, ETR, and qP by 17.78%, 17.08%, and 11.11%, respectively. The γ-PGA treatment also significantly improved ФPSII, ETR, and qP by 13.95%, 12.26%, and 9.68%, respectively. As a result, Glu and γ-PGA priming enhanced the efficiency of the photochemical reaction, being consistent with a more robust photosynthetic process as indicated by the improved gas exchange parameters. However, it was notable that NPQ was significantly reduced by 14.81% and 11.11% in Glu- and γ-PGA-primed heat-stressed plants, respectively, as compared to the control.

### 2.5. Transcriptome Changes in Response to Glu and γ-PGA Priming

**Figure 4:**
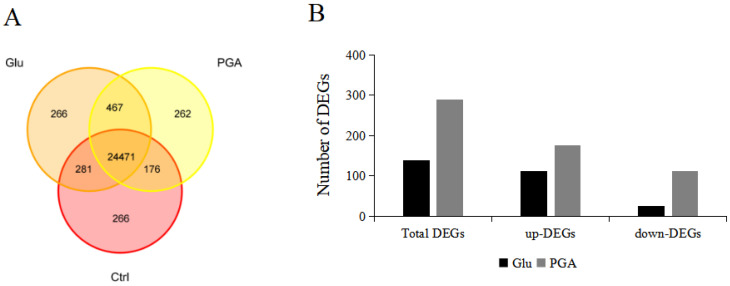

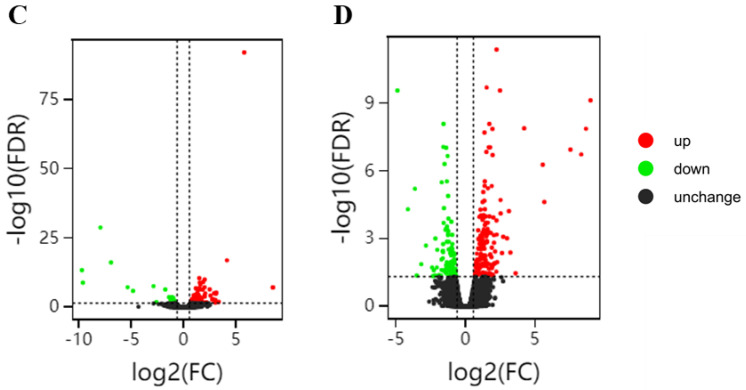
(**A**) Venn diagram of expressed genes in Glu- and γ-PGA-primed plants under heat stress; (**B**) number of differential genes; (**C**) the volcanic plot of Glu DEGs relative to Ctrl.; (**D**) the volcanic plot of γ-PGA DEGs relative to Ctrl. X-axis depicts fold change in gene expression where, the smaller the value, the higher the expression relative to Ctrl. Y-axis denotes false discovery rate where, the bigger the value, the smaller the ratio of the number of false positives to the number of total positives. Red and green dots represent significantly up-regulated and down-regulated DEGs, respectively. Black dot indicates genes that were not differentially expressed.

In order to explore the Glu- and γ-PGA-priming-induced differentially expressed genes (DEGs), some of which may be involved in the preadaptation to heat stress, we performed RNA-seq analysis using Glu- and γ-PGA-primed heat-stressed Chinese cabbage along with a control. A total of 80.92 Gb clean reads were obtained. All clean data were then de novo assembled, and assembly of transcript results was evaluated. Approximately 82.91% of control, 83.27% of Glu, and 84.69% of γ-PGA assembled reads were aligned uniquely with *Brassica rapa* ssp. *pekinensis* reference genome (version 3.0). A total of 24,752 genes between control and Glu, 24,647 genes between control and γ-PGA, and 24,471 genes among control, Glu, and γ-PGA were commonly expressed (Figure 4A). Among the identified genes, 139 and 289 DEGs were specifically expressed in Glu and γ-PGA compared to the control, respectively (Figure 4B).

### 2.6. KEGG Pathway Enrichment Analysis

**Figure 5:**
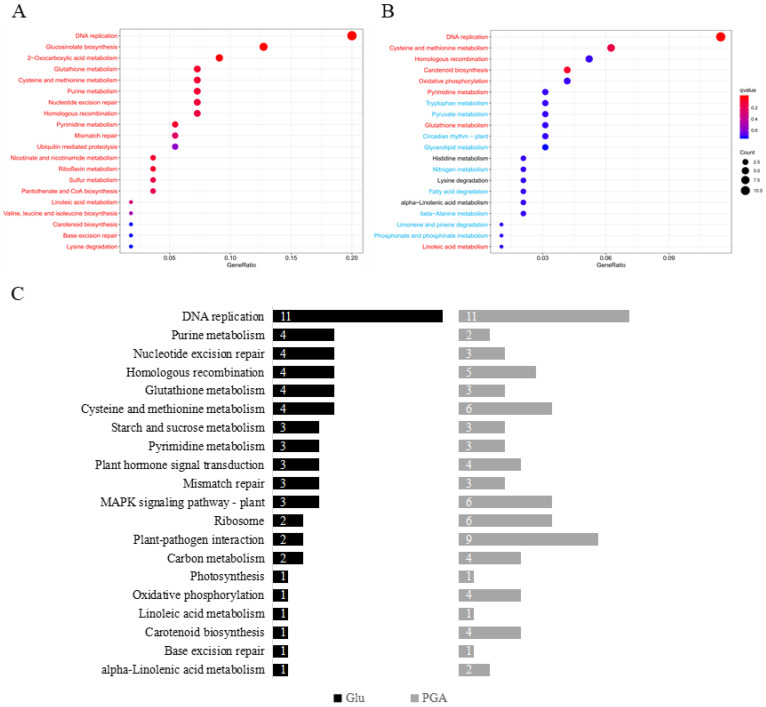
KEGG classifications of DEGs in different groups. The vertical axis represents the pathway name, and the horizontal axis represents the gene number ratio of significant DEGs to the total genes in a given KEGG pathway. The size of the dots indicated by “Count” represents the number of DEGs in the pathway, and the color of the dots corresponds to the different q-value ranges. (**A**) Glu relative to Ctrl.; (**B**) γ-PGA relative to Ctrl.; (**C**) Glu and γ-PGA commonly expressed relative to Ctrl. Each figure showed top 20 pathways with q-values. Red and blue letters indicate more than 50% up-regulated and down-regulated DEGs out of a total number of DEGs, respectively. Black letter indicates each 50% of up- and down-regulated DEGs.

To identify the biological pathways associated with sets of genes expressed in the condition of Glu- and γ-PGA-primed heat stress, we conducted KEGG enrichment analysis of DEGs in Glu and γ-PGA relative to the control (Figure 5). As compared to the control, Glu induced significant up-regulation of 61 DEGs and down-regulation of 12 DEGs (Figure 5A; Table S1), whereas γ-PGA triggered significant up-regulation of 54 DEGs and down-regulation of 38 DEGs (Figure 5B; Table S2). Based on the q values and GeneRatio, 20 of the most enriched KEGG pathways were selected. The KEGG analysis between the control and Glu (Figure 5A) showed that the most abundantly regulated pathways were DNA replication (eleven genes), glucosinolate biosynthesis (seven genes), and 2-oxocarboxylic acid metabolism pathway (five genes), followed by glutathione metabolism (four genes), cysteine and methionine metabolism (four genes), purine metabolism (four genes), nucleotide excision repair (four genes), and homologous recombination (four genes). The most abundantly regulated pathways in γ-PGA treatment were DNA replication (eleven genes), plant–pathogen interaction (nine genes), followed by cysteine and methionine metabolism (six genes), *MAPK* signaling pathway (six genes), plant–pathogen interaction (six genes), homologous recombination (five genes), carotenoid biosynthesis (four genes), and oxidative phosphorylation (four genes) (Figure 5B). Notably, genes involved in the DNA repair system, such as homologous recombination, nucleotide excision, and base excision repair, were up-regulated in both Glu and γ-PGA. The genes involved in glucosinolate biosynthesis were up-regulated only by Glu (Table S1, Figure 5A,B).

### 2.7. Validation of DEGs by qRT-PCR

**Figure 6:**
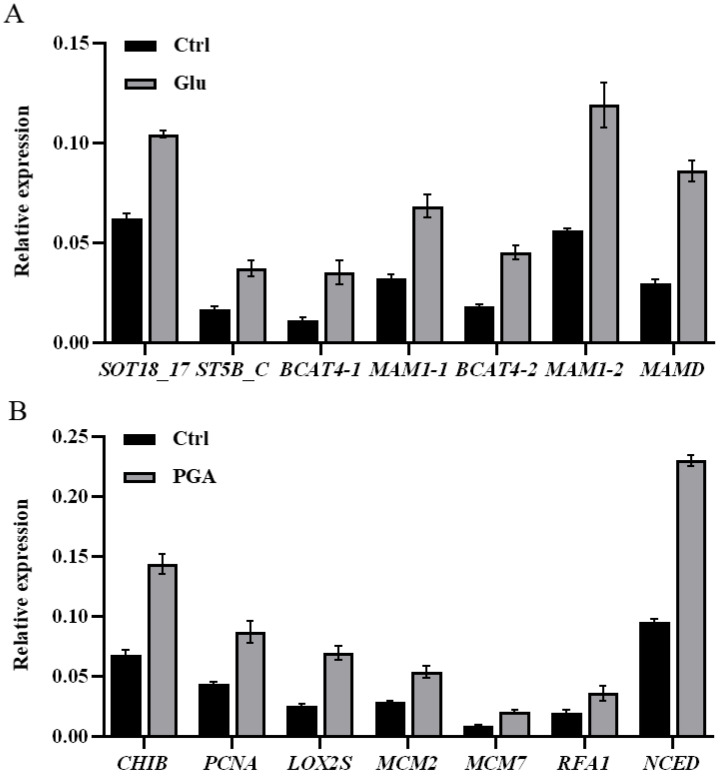
Gene expression pattern verification by qRT-PCR. (**A**) Gene expression of Glu vs. Ctrl. (**B**) Gene expression of γ-PGA vs. Ctrl. *CHIB* (*BraA03g035760.3.1C*), *PCNA* (*BraA09g064370.3.1C*), *LOX2S* (*BraA02g016330.3.1C*), *MCM2* (*BraA10g007700.3.1C*), *MCM7 (BraA09g002250.3.1C*), *RFA1* (*BraA03g003290.3.1C*), *NCED* (*BraA01g038240.3.1C*).

In order to validate our transcriptome data, 14 DEGs involved in the KEGG pathways of Glu and γ-PGA treatment groups were randomly selected for qRT-PCR (Figure 6). All the DEGs showed significantly increased expression, among which were seven glucosinolate biosynthesis genes in the Glu priming group. The isopropylmalate/methylthioalkylmalate dehydrogenase encoded by *MAMD* is involved in methionine chain elongation in the initial step of aliphatic GSLs biosynthesis [36]. The methylthioalkylmalate synthase encoded by *MAM* catalyzes exclusively the condensation reactions of both the first and second methionine carbon chain elongation [37]. The methionine aminotransferase encoded by *BCAT4* is involved in the chain elongation pathway in the biosynthesis of methionine-derived GSLs [38]. The aliphatic desulfoglucosinolate sulfotransferase encoded by *ST5B_C* and *SOT18_17* catalyzes the last step in the biosynthesis of aliphatic GSLs core structures [39]. Seven genes of the γ-PGA priming group also showed significantly increased expression. The chitinase-like protein encoded by *CHIB* was reported to be involved in Chinese cabbage plant resistance against biotic stresses [40]. A chloroplast lipoxygenase encoded by *LOX2S* is required for the wound-induced synthesis of jasmonic acid (JA) in leaves [41]. The 9-cis-epoxycarotenoid dioxygenase encoded by *NCED* is a rate-limiting enzyme that catalyzes the first step of abscisic acid biosynthesis from carotenoid [42]. The proteins encoded by *PCNA*, *MCM2*, *MCM7*, and RFA1 play crucial roles in DNA replication and DNA repair [43,44,45]. The results of qRT-PCR showed a similar expression profile to those from the RNA-seq data (Table S4). These results independently confirmed that high coverage of transcriptome data is reliable.

## 3. Discussion

### 3.1. Molecular Aspects of Acquired Thermotolerance Conferred by Exogenous Glu and γ-PGA

Since heat causes damage to a range of cellular components, a large number of different multigenic protective pathways and a complex regulatory network are expected to be involved in the acquisition of thermotolerance. The fact that Glu and γ-PGA triggered changes in expression profiles of fourteen different categories of KEGG pathway metabolisms in Chinese cabbage leaves suggests that they may induce heat tolerance by modulating the plant physiological status for preadaptation to stress-acclimating processes. Above all, heat stress is known to not only inhibit almost all DNA repair systems but also to directly induce single- and double-stranded DNA breaks [46]. Our KEGG analysis showed that DNA replication, metabolism of purine and pyrimidine, and all major types of DNA damage repair, including homologous recombination, nucleotide excision, base excision, and mismatch repair, were commonly up-regulated by both Glu and γ-PGA priming to maintain genomic integrity during replication stress. One notable DEG is the RPA1 gene (BraA03g003290.3.1C) encoding replication protein A 70 kDa DNA-binding subunit B (Tables S1 and S2) that plays essential roles in almost all DNA metabolic pathways, including DNA replication, transcription, recombination, DNA damage surveillance and recognition, cell-cycle checkpoints, and in all major types of DNA repair, including base excision, nucleotide excision, mismatch, and double-strand break repair [43]. Hence, exogenous Glu and γ-PGA activate the repairing system of chromatin structural changes as one of the primary impacts caused by heat stress [47]. To combat free radicals that are explosively generated during heat stress, glutathione S-Transferases (GSTs) genes associated with metabolism of glutathione were also up-regulated by both Glu and γ-PGA priming (Tables S1 and S2). GSTs were identified as stress response proteins that accumulated in response to a wide range of biotic and abiotic stresses since they are a family of Phase II detoxification enzymes that function to protect cellular macromolecules from attack by reactive electrophiles [48].

In addition to the role in detoxification, conjugation of glutathione (GSH) catalyzed by GST is required for biosynthesis of glucosinolates (GSLs), which are primarily found in plants of the genus Brassica and involved in plant defense [49]. It has been shown that an *Arabidopsis thaliana* mutant with GSLs metabolic defects shows a decrease in the levels of heat shock stress protein Hsp90 and a decrease in tolerance to high temperatures [50]. Thus, GSLs may contribute to heat tolerance by modulating the plant physiological status in a similar way to stress-acclimating processes [51]. Our KEGG analysis along with RT-PCR showed that the biosynthesis pathways for aliphatic GSLs were up-regulated in Glu-treated plants under heat stress. *Arabidopsis thaliana* exposed to high salt stress was also shown to increase the production of short-chain aliphatic GLSs and decrease the production of indolic GSLs [51], suggesting their role in adaptation of the plant in response to environmental stresses besides their prominent role involved in defense mechanism. Along this line, it was reported that GSLs did not display any free radical scavenging activity but showed ferrous ion (Fe+2)-chelating ability [52]. Unlike Glu, γ-PGA did not induce GSLs genes, suggesting that GSLs are not essential for the acquisition of thermotolerance. Although the reason for this difference remains unclear, this could be due to the priming-chemicals-dependent variation of GSLs accumulation depending on the tissue type. A study by Troufflard et al. [53] showed that A. thaliana accumulated more GSLs in the roots than in the shoots in response to abiotic stress of potassium deficiency. Although the physiological role of GSLs in response to abiotic stress is still unknown, environmental factors may alter GSLs composition and profile [51].

Apart from GSLs biosynthesis, Glu induced not only biosynthesis of the 2-oxo acids of valine, leucine and isoleucine, from which GSLs can be derived but also sulfur metabolism. GSLs are sulfur-rich compounds that are composed of thiohydroxymates carrying an S-linked β-glucopyranosyl residue and an N-linked sulfate bearing an aliphatic, indolic, or aromatic group side chain derived from amino acids. According to the KEGG 2-oxocarboxylic acids metabolism, biosynthesis of valine, leucine, and isoleucine is derived from 2-oxoisovalerianic acid, 2-oxocarpronic acid, and 2-oxobutyric acid, respectively. Thus, Glu treatment appears to bring about the coordinated up-regulation of GSLs, 2-oxocarboxylic acids, and sulfur metabolism pathways.

Carotenoids are essential photosynthetic pigments [54], which mainly exist in plastids of both photosynthetic and non-photosynthetic plant tissues [55]. It has two main functions: (i) harvesting light energy followed by transferring the excitation energy directly to chlorophyll molecules for photosynthesis, and (ii) protecting chlorophyll and the surrounding cell from oxidative damage by absorbing the excess light energy and dissipating it as heat. Chlorophylls often generate toxic ROS, which cause diverse cellular damage, and they are particularly prone to generating such free radicals under high light or temperature conditions. Carotenoids could remove ^1^O_2_ and prevent the formation of ^1^O_2_ by reacting with 3Chl* and excited chlorophyll, thus protecting the photosynthetic apparatus [56]. Our study showed that application of exogenous Glu and γ-PGA significantly increased carotenoid content under heat stress conditions (Table 3). RNA-seq analysis revealed that expression of 9-cis-epoxycarotenoid dioxygenase (*NCED*) transcript was significantly increased in γ-PGA-treated plants under heat stress conditions, although it was not affected in Glu-treated plants. RT-PCR analysis was also performed to further confirm it. In Arabidopsis, the carotenoid cleavage reaction catalyzed by *NCED* as the first step of ABA biosynthesis was induced by drought stress [57]. Accordingly, γ-PGA appeared to improve the thermotolerance through the biosynthesis of both carotenoids and ABA.

ABA is known to regulate expression of pathogenesis-related (PR) proteins, which are significantly induced by multiple biotic and abiotic stresses [58]. Our KEGG analysis indicated that both exogenous Glu and γ-PGA triggered up-regulation of transcription factors involved in regulating stress responses in the category of plant–pathogen interaction (Tables S1 and S2). Interestingly, the gene (*BraA03g030100.3.1C*) encoding abscisic acid receptor, which is a core regulatory component of ABA signaling networks in plants [59], was down-regulated in Glu-treated plants, while it was not affected in γ-PGA-treated plants. Consequently, Glu and γ-PGA priming appears to trigger a change in plant physiological and biochemical status in a different manner for preadaptation to stress-acclimating processes. Although ABA-mediated stress responses differ between Glu- and γ-PGA-treated plants, the lipoxygenase gene (*BraA02g016330.3.1C*) required for the synthesis of jasmonic acid that plays an active role in regulating plant development and abiotic stress responses [41] was up-regulated in both Glu- and γ-PGA-treated plants.

### 3.2. Exogenous Glu and γ-PGA Improve Photosynthesis in Chinese Cabbage Seedlings under Heat Stress

It is well known that heat stress substantially decreases Pn, Tr, Gs, and Ci, leading to severe inhibition of photosynthesis efficiency [60,61]. We found that foliar applications of Glu and γ-PGA significantly increased such gas exchange parameters of Chinese cabbage leaves grown under high-temperature stress. This result confirmed the previous finding that the ability to sustain leaf gas exchange under heat stress is directly correlated with thermotolerance in all plant species [47].

Apart from the gas exchange, chlorophyll fluorescence measurement is also a valuable analytical tool for evaluating photosynthetic performance of plants under environmental stresses [62,63]. In our study, Glu and γ-PGA pre-treatment resulted in decreased NPQ and increased qP coefficient compared to the control, indicating that Glu and γ-PGA promoted photochemical energy transfer and inhibited non-photochemical energy dissipation processes of PSII to sustain high photochemical reaction efficiency. Consistent with the increased qP value, Glu- and γ-PGA-primed plants had higher values of Fv/Fo, Fv/Fm, ETR, and ΦPSII than the non-primed control plants. Fv/Fm has been generally used to estimate the extent of heat-induced damage to PSII since heat stress decreases Fv/Fm, and the average maximum Fv/Fm value ranges from 0.79 to 0.84 in many plant species [35]. In this work, both Glu- and γ-PGA-primed plants had approximately 0.80 of Fv/Fm, suggesting that the photosynthetic performance supported by Glu and γ-PGA treatment was comparable to that of the unstressed plants. The ratio Fv/Fo is known to be more sensitive than Fv/Fm at stress conditions since it expresses the efficiency of the water-splitting complex on the donor side of PSII, which is the most responsive component in the photosynthetic electron transport chain [64]. Glu-primed plant exhibited a significantly higher Fv/Fo ratio than γ-PGA-primed plant, being consistent with the fact that Glu application led to significantly higher biomass and chlorophyll than γ-PGA treatment.

Chlorophyll (Chl) absorbs light energy and transfers excited electrons to adjacent pigments to initiate charge separation in the photosystem reaction centers, and, thus, maintaining stable chl levels is critical for efficient photosynthesis [65]. Foliar application of Glu and γ-PGA significantly increased the content of chl *a* and *b* when compared to the control. This is in agreement with the fact that chlorophyll content is positively correlated with growth and biomass of plants [60,66]. The reaction center core complexes of PSI and PSII contain only chl *a*, whereas the peripheral light-harvesting antenna complex *(LHC*) embedded in the thylakoid membranes consists of chl *a*, chl *b*, and carotenoid [67]. Because chl *b* is present exclusively in *LHCI* and *LHCII*, the lower chl *a*/*b* ratio in the Glu- and γ-PGA-primed plants indicates that the chemical priming increased amounts of LHC proteins relative to the reaction center complexes. This light-harvesting mode typically occurs as an adaptation to the low-intensity light conditions (shaded leave), where low energy dissipation is induced with high electron transport rate [68]. However, unlike the shaded leaves containing the declined amounts of carotenoids in many plant species [69], Glu- and γ-PGA-primed plants grown under heat stress had increased amounts of carotenoids as compared to the control. Carotenoids have two main functions as an accessory light-harvesting pigment and a radical-scavenging antioxidant: harvesting light energy followed by transferring the excitation energy directly to chlorophyll molecules for photosynthesis and protecting chlorophyll and the surrounding cell from oxidative damage by absorbing the excess light energy and dissipating it as heat [70,71]. Despite the involvement of carotenoids with many chlorophylls in the NPQ process [72,73], decreases in NPQ relative to the control were noted in Glu- and γ-PGA-primed plants that had higher contents of chlorophyll and carotenoids (Table 3). This means that Glu and γ-PGA may strengthen the light harvesting state and lessen the quenching state of LHC. This would increase the number of photons available for photosynthesis to support the production of more protective metabolites and enzymes to combat excess ROS generated in various cellular compartments by heat stress. Glu treatment led to the higher ratio of chlorophyll to carotenoid than γ-PGA application, which was positively correlated with our finding that Glu application led to significantly higher biomass than γ-PGA treatment. Since carotenoids are essential photosynthetic pigments, synthesis of carotenoids should be coordinately controlled with chlorophyll synthesis for optimal photosynthesis [74].

Unlike the KEGG carotenoid biosynthesis pathway up-regulated by Glu and γ-PGA, KEGG categories of photosynthesis and chlorophyll metabolism in both Glu- and γ-PGA-primed plants were absent despite the increases in chlorophyll content and photosynthetic activity. This implicates that exogenous Glu and γ-PGA may stimulate chlorophyll synthesis at either the post-transcriptional or post-translational level. Along this line, post-translational regulators were postulated to promote chlorophyll homeostasis by adjusting the balance between chlorophyll biosynthesis and breakdown during leaf development [75].

### 3.3. Exogenous Glu and γ-PGA Mitigate the Growth Inhibition Caused by Heat Stress in Chinese Cabbage Seedlings

When plants are exposed to heat, excessive ROS is rapidly produced to cause damage to DNA, proteins, lipids, carbohydrates, membranes, and organelles [76,77]. In this work, we evaluated the impact of Glu and γ-PGA priming on the levels of MDA, EL, proline, and antioxidant enzyme activities in *B. rapa* seedlings under heat treatment, all of which greatly vary depending on the amount of ROS and have been used as tolerance indicators for abiotic stresses [6]. Glu and γ-PGA substantially reduced the oxidative stress levels of ion leakage, MDA, and superoxide radical as compared to the control, which occurred in parallel with the enhanced activities of SOD, APX, CAT, and POD enzymes capable of detoxifying ROS. This result indicates that Glu and γ-PGA can effectively enhance the removal of excessive ROS produced under heat stress. The higher activity levels of such antioxidant enzymes would protect the integrity of multiple intracellular compartments, including the photosynthetic apparatus, to sustain a higher Pn under heat stress. In addition, Glu and γ-PGA priming markedly enhanced the content of proline, which plays four major roles during abiotic stresses by acting as an osmolyte, a metal chelator, an antioxidant ROS scavenging molecule, and a redox signaling molecule [31]. Similar results were previously obtained from Glu-primed rice seedlings under cadmium stress, γ-PGA-primed oilseed rape seedlings under cold and drought stress, and γ-PGA-primed canola seedlings under salt stress [23,25,78,79].

Despite the significant difference in the levels of MDA, superoxide radical, and proline between Glu and γ-PGA treatments, no significant differences in ROS scavenging enzyme activity levels were observed between Glu- and γ-PGA-primed heat-stressed plants, both of which had higher enzyme activities than the unprimed heat stress control plant. This non-linear relationship between the levels of oxidative stress biochemical markers and antioxidant enzyme activities could be attributed to the hyperbolic relationship between the rate of enzymatic reaction and the substrate concentration of ROS, which typically occurs in enzyme-catalyzed reactions. Our study also showed that there were negative correlations between biomass and levels of MDA and EL, as well as a positive correlation between biomass and proline content. Heat stress alone without chemical priming resulted in much lower levels of antioxidant enzymes and proline, indicating that the uncontrolled ROS level triggered by 45 °C heat treatment caused oxidative damage to the integrity of biomolecules and thus decreased the enzyme activity level. Taken together, our results showed the importance of ROS scavenging in the acquired acclimation response to excess heat, during which Glu and γ-PGA assisted in maintaining steady-state tolerant levels of ROS in *B. rapa* seedlings through antioxidation and osmoprotection to enhance plant functionality at the whole plant level.

## 4. Materials and Methods

### 4.1. Plant Materials and Treatments

The Chinese cabbage variety of ‘Beijing No.3’ was used in this study. Seeds were sown in a soil mix of peat, vermiculite, and perlite in a 3:2:1 ratio and then kept in the artificial climate equipment with 65% relative humidity and 600 μmol∙m^−2^∙s^−1^ maximum light intensity at 24 °C during the day and 8 h at 22 °C during the night. After 10 days, plants in cotyledon stage were transplanted to plastic pots (7 cm in diameter and 7 cm in height) under the same conditions. When the third leaf of Chinese cabbage seedlings was fully developed, the leaves were sprayed daily with H_2_O as control, Glu (2.0 mM, Shanghai Yuanye Bio-Technology Co., Ltd., Shanghai, China), or γ-PGA (20 mg·L^−1^, 2 kDa, Shanghai Yuanye Bio-Technology Co., Ltd., Shanghai, China) for 5 days. All seedlings were then subjected to heat stress (45 °C in 16-h light/35 °C in 8-h dark) for 5 days.

### 4.2. Measurement of Biomass and Physiological Parameters

A ruler was used to measure the plant height, as well as the length and width of the third leaf of seedlings. Hypocotyl diameters were measured with a digital vernier calliper. The fresh weight and dry weight were estimated on an electronic balance. For drying, the samples were placed in an oven at 75 °C to a constant weight.

Malondialdehyde content was determined according to the method [80]. Proline content was measured by acidic ninhydrin colorimetric assay [81]. The leaf electrolyte leakage assay was performed following the method [82]. Activities of superoxide dismutase, peroxidase, catalase, superoxide radical, and ascorbate peroxidase were determined using the corresponding kits R21883, R30312, R21885, R30343 (Shanghai Yuanye Bio-Technology Co., Ltd., Shanghai, China), and A123-1-1 (Nanjing Jiancheng Bioengineering Institute, Nanjing, China), respectively.

### 4.3. Measurements of Gas Exchange and Chlorophyll Fluorescence Parameters

Leaf gas exchange (net photosynthetic rate, Pn; stomatal conductance, Gs; intercellular CO_2_ concentration, Ci; transpiration rate, Tr) was measured with LI-6800 portable photosynthesis system (LI-6800F; LI-COR, Beijing Ecotek Technology Co., Ltd., Beijing China) on the third leaf of Chinese cabbage seedlings from 9:00 a.m. to 12:00 a.m. on sunny days. Irradiance level was set at 800 μmol photons·m^−2^·s^−1^. CO_2_ concentration was set at 380 μmol·mol-1 with 25 °C air temperature and 60% relative humidity.

Plants were dark-adapted for 20 min at 25 °C before measuring the chlorophyll fluorescence parameters with a portable spectrometer (PAM-2500, Heinz Walz GmbH, Germany). The maximum potential photochemical efficiency of PSII (Fv/Fm), electron transfer rate of PSII (ETR), non-photochemical fluorescence quenching (NPQ), and photochemical fluorescence quenching (qP) revealed the actual quantum efficiency of PSII (ΦPSII) and were calculated according to previous reports [83]. Fv/Fm = (Fm − Fo)/Fm, where Fv, Fm, and Fo are the variable, maximum, and minimum fluorescence level in the dark-adapted state, respectively; ΦPSII = (Fm′ − Ft)/Fm′; NPQ = (Fm − Fm′)/Fm′; qP = (Fm′ − Ft)/(Fm′ − Fo′), where Fm′ and Fo′ are maximum and minimum fluorescence levels in the light-adapted state, respectively, whereas Ft is a steady-state level of fluorescence level during actinic light illumination.

### 4.4. SPAD and Spectrophotometric Measurements of Photosynthetic Pigments

Chlorophyll meter SPAD-502Plus (Konica Minolta Sensing, Inc., Tokyo, Japan) was used to estimate the relative amounts of chlorophyll (chl) at a position 2/3 of the distance from the leaf base of the uppermost fully expanded leaf.

The absolute concentrations of photosynthetic pigments (chlorophyll *a*, chl *a*; chlorophyll *b*, chl *b*; carotenoid, Car) were determined using fresh leaves according to the previous method [64]. Briefly, 0.5 g of Chinese cabbage leaves were soaked in extraction solution (10 mL of 80% acetone and 5 mL of 95% ethyl alcohol) in the dark for 24 h. The absorbance of the extract was recorded at 440 nm, 663 nm, and 645 nm in a UV spectrophotometer (Shimadzu UV-1800, Tokyo, Japan).

### 4.5. Total RNA Extraction and Quality Control

Total RNA was extracted from the leaves using NEBNext UltraTM RNA Library Prep Kit for Illumina (NEB, Ipswich, MA, USA) according to the manufacturer’s instructions. RNA degradation and contamination were monitored using 1% agarose gels. RNA purity was evaluated using NanoDrop 2000 (Thermo Fisher Scientific, Waltham, MA, USA). RNA integrity was evaluated using the RNA Nano 6000 Assay Kit of the Agilent Bioanalyzer 2100 system (Agilent Technologies, Santa Clara, CA, USA).

### 4.6. RNA Sequencing and RNA-Seq Data Analysis

A total amount of 1 μg RNA per sample was used for transcriptome sequencing. RNA-seq libraries were constructed using the NEBNext UltraTM RNA Library Prep Kit (NEB, USA). Three biological replicates were performed for each treatment. RNA sequencing and primary bioinformatics analysis were performed by BMKCloud platform (http://en.biomarker.com.cn/biocloud accessed on 30 January 2022). Kyoto Encyclopedia of Genes and Genomes (KEGG) is a database resource for understanding high-level functions and utilities of the biological system (http://www.genome.jp/kegg/ accessed on 30 January 2022).

### 4.7. Transcriptomic and sRNA Data Validation by qRT-PCR

RNA sequencing and RNA-seq data analysis 2.7 High quality RNA samples from leaves of Ctrl, Glu, and γ-PGA seedlings were used for qRT-PCR to measure the transcriptional levels of different genes. The cDNA synthesis and qRT-PCR reactions were carried out using a HiScript^®^ III All-in-one RT SuperMix Perfect Kit on a qTOWER3G Real-Time PCR Detection System (Analytik, Jena, Germany). The house-keeping gene BrActin was used as a reference gene for quantitative validation of the expression data. The gene-specific primers designed for the nine selected genes are listed in Table S4. The reaction system was as follows: 10 μL of 2× qPCR mix, 0.5 μL of 10 μmol gene primer, 2 μL cDNA, and 7 μL of ddH2O. The PCR cycling conditions comprised an initial polymerase activation step of 95 °C for 30 s, followed by 40 cycles of 95 °C for 8 s and 60 °C for 6 s, and a dissociation curve analysis was carried out from 60 °C to 95 °C at the end of a PCR experiment. The 2^−ΔΔCT^ method was used to calculate the fold change of transcript level [84]. Data were analyzed from three independent sets of biological replicates with three technical replicates for each.

### 4.8. Statistical Analyses

For all treatments, five biological replications were performed. The results were expressed as mean ± standard error, and Graph Pad prism 9.0.0. software was used to draw the graphs and analyze the data. All data were subjected to analysis of variance for a factorial experiment in a completely randomized design. Statistically significant differences between means were determined at *p* < 0.05 using Tukey’s HSD (honestly significant difference) test.

## 5. Conclusions

**Figure 7:**
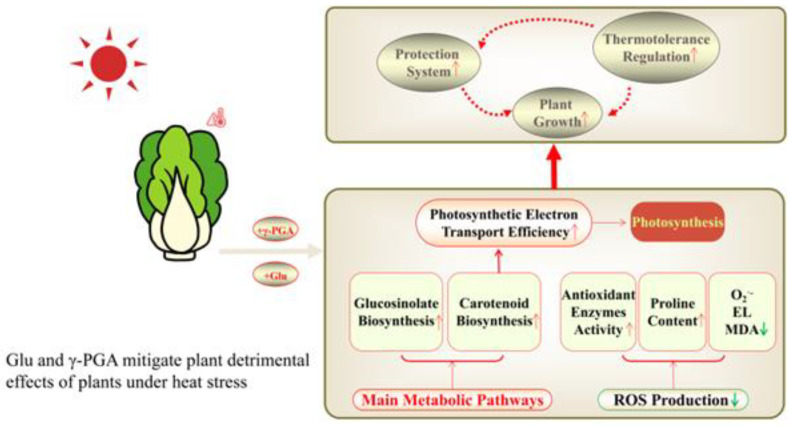
Schematic summary of Glu- and γ-PGA-primed physiological status for the preadaptation to heat stress in Chinese cabbage seedlings. The solid arrows represent enhancement, whereas the dotted arrows represent inhibition.

Our study demonstrated that exogenous application of Glu or γ-PGA could alleviate the damage caused by heat stress in Chinese cabbage and improve the growth parameters and physiological characteristics (Figure 7). Comparative phenotypic, physiological, and RNA-seq analysis revealed that the improvement in heat tolerance of Chinese cabbage by exogenous application of Glu or γ-PGA may be attributed to the increase in carotenoid, chlorophyll content, and photosynthesis capacity, reduction in ROS, maintenance of PSII activity, improvement in osmolytes accumulation, and activation of antioxidant enzymes and all major DNA repair system types. The biosynthesis of glucosinolates contributes to the heat tolerance of Chinese cabbage, but it is not essential for thermotolerance. Both Glu and γ-PGA have significant potential for the preadaptation of Chinese cabbage seedlings to heat stress, with Glu being more effective than γ-PGA. Further research at the level of proteomics and metabolomics is necessary to reveal the underlying causes of Glu- and γ-PGA-primed changes in physiological and metabolic status that can contribute to more rapid systemic acquired acclimation to heat stress. Previous findings indicated that γ-PGA was effective for improvement in enzyme activity, as well as that of enzyme stability for thermal denaturation [85,86]. The beneficial effects of seed priming on their resistance to environmental stresses were documented [87]. Further research on seed priming with Glu and γ-PGA and the duration of chemical priming efficacy is necessary for their economical and practical use in the agricultural field.

## Figures and Tables

**Table 1:** Effects of Glu and γ-PGA on the vegetative growth of Chinese cabbage under heat stress.

Treatment	Fresh Weight (g∙Plant^−1^)	Dry Weight (g∙Plant^−1^)	Plant Height (cm)	Hypocotyl Diameter (mm)	Leaf Length (cm)	Leaf Width (cm)
Ctrl	3.51 ± 0.17 c	0.33 ± 0.02 b	12.60 ± 0.36 b	2.25 ± 0.06 b	11.63 ± 0.35 b	4.67 ± 0.31 b
Glu	5.18 ± 0.19 a	0.50 ± 0.02 a	16.10 ± 0.32 a	2.56 ± 0.06 a	15.76 ± 0.28 a	6.68 ± 0.29 a
PGA	4.82 ± 0.16 b	0.46 ± 0.02 a	15.93 ± 0.33 a	2.46 ± 0.05 a	15.44 ± 0.25 a	6.13 ± 0.19 a

Note: seedlings were foliar sprayed with water (Ctrl), Glu (Glu), or γ-PGA (PGA), followed by exposure to high temperature. Each value represents the mean ± SD. Different lowercase letters of the same column indicate significant difference at *p*  <  0.05.

**Table 2:** Effect of Glu and γ-PGA on the antioxidant enzyme activity and superoxide radical production under heat stress.

Treatments	Antioxidant Enzyme (U·g^−1^ FW)				Superoxide Radical Content (μmol·g^−1^ FW)
	SOD	APX	CAT	POD	O_2_^·−^
Ctrl	255.31 ± 0.32 b	0.56 ± 0.13 b	333.44 ± 0.12 b	32.29 ± 1.45 b	23.39 ± 0.82 a
Glu	303.98 ± 0.61 a	0.72 ± 0.29 a	447.44 ± 0.21 a	46.97 ± 1.93 a	15.65 ± 0.19 c
PGA	289.29 ± 0.75 a	0.71 ± 0.28 a	444.19 ± 0.18 a	44.90 ± 1.93 a	18.12 ± 0.16 b

Note: seedlings were foliar sprayed with water (Ctrl), Glu (Glu), or γ-PGA (PGA), followed by exposure to high temperature. Each value represents the mean  ±  SD. Different lowercase letters of the same column indicate significant difference at *p*  <  0.05.

**Table 3:** Effects of Glu and γ-PGA on photosynthetic pigment of Chinese cabbage under heat stress.

Pigment	Treatments		
	Ctrl	Glu	PGA
SPAD	29.13 ± 0.54 b	32.73 ± 0.46 a	32.23 ± 0.19 a
chl *a* (mg·g^−1^ FW)	1.54 ± 0.03 b	2.13 ± 0.05 a	1.88 ± 0.03 a
chl *b* (mg·g^−1^ FW)	0.45 ± 0.01 c	0.69 ± 0.03 a	0.57 ± 0.07 b
total chl (mg·g^−1^ FW)	1.99 ± 0.04 c	2.83 ± 0.05 a	2.45 ± 0.02 b
carotenoid (mg·g^−1^ FW)	0.25 ± 0.05 b	0.33 ± 0.06 a	0.31 ± 0.04 a
chl *a*/*b*	3.39 ± 0.04 b	3.06 ± 0.08 a	3.29 ± 0.10 b
chl/car	8.07 ± 0.19 ab	8.54 ± 0.30 a	7.82 ± 0.16 b

Note: seedlings were foliar sprayed with water (Ctrl), Glu (Glu), or γ-PGA (PGA), followed by exposure to high temperature. Each value represents the mean ± SD. Different lowercase letters of the same column indicate significant difference at *p*  <  0.05.

**Table 4:** Effects of Glu and γ-PGA on chlorophyll fluorescence parameters in Chinese cabbage under heat stress.

Treatment	Fv/Fo	Fv/Fm	ETR	ΦPSII	NPQ	qP
Ctrl	3.15 ± 0.06 c	0.77 ± 0.04 b	31.70 ± 0.12 c	0.37 ± 0.01 b	0.27 ± 0.01 a	0.56 ± 0.02 b
Glu	4.08 ± 0.04 a	0.80 ± 0.04 a	38.23 ± 1.14 a	0.45 ± 0.03 a	0.23 ± 0.02 b	0.63 ± 0.01 a
PGA	3.74 ± 0.04 b	0.79 ± 0.01 a	36.13 ± 0.54 b	0.43 ± 0.01 a	0.24 ± 0.02 ab	0.62 ± 0.01 a

Note: seedlings were foliar sprayed with water (Ctrl), Glu (Glu), or γ-PGA (PGA), followed by exposure to high temperature. Each value represents mean ± SD. Different lowercase letters of the same column indicate significant difference at *p*  <  0.05.

## Data Availability

The data presented in this study are available in this manuscript.

## Supplementary Materials

The following supporting information can be downloaded at: https://www.mdpi.com/article/10.3390/ijms231911671/s1.
