# Supplementary material for: Glutamic Acid and Poly-γ-glutamic Acid Enhanced the Heat Resistance of Chinese Cabbage (Brassica rapa L. ssp. pekinensis) by Improving Carotenoid Biosynthesis, Photosynthesis, and ROS Signaling
Source: Int J Mol Sci. 2022 Oct 1;23(19):11671. doi: 10.3390/ijms231911671 (PMC9570168; doi:10.3390/ijms231911671)
Supplement: Supplementary file 1 [file ijms-23-11671-s001.zip › Supplemental Tables S1 and S2.pdf]

http://brassicadb.cn/#/GeneSequence/

Table S1. KEGG enrichment analysis of DEGs in Glu relative to Ctrl.

KEGG ID: ko03030 **DNA replication**

10 Up-DEGs

- BraA02g033700.3.1C Brassica rapa DNA replication licensing factor MCM3: component of the MCM2-7 complex (MCM complex) that may function as a DNA helicase and which is essential to undergo a single round of replication initiation and elongation per cell cycle in eukaryotic cells. MCM2-7 complex is activated to unwind the double stranded DNA and plays an important role in DNA replication forks elongation
- BraA03g003290.3.1C Brassica rapa replication protein A 70 kDa DNA-binding subunit B (RPA1B): DNA unwinding involved in DNA replication, double-strand break repair via homologous recombination, meiotic cell cycle, nucleotide-excision repair, telomere maintenance via telomerase.
- BraA03g042570.3.1C Brassica rapa DNA replication licensing factor MCM5
- BraA03g043620.3.1C Brassica rapa DNA replication licensing factor MCM4
- BraA06g043190.3.1C Brassica rapa DNA replication licensing factor MCM3
- BraA06g044160.3.1C Brassica rapa DNA replication licensing factor MCM6
- BraA07g017290.3.1C Brassica rapa DNA polymerase alpha catalytic subunit: plays an essential role in the initiation of DNA synthesis by extending the primers.
- BraA09g002250.3.1C Brassica rapa DNA replication licensing factor MCM7
- BraA09g064370.3.1C Brassica rapa proliferating cell nuclear antigen (PCNA): a processivity factor for DNA polymerase δ in eukaryotic cells and is essential for replication. In response to DNA damage, this protein is ubiquitinated and is involved in the RAD6-dependent DNA repair pathway. PCNA is important for both DNA synthesis and DNA repair. Essers J, Theil AF, Baldeyron C, van Cappellen WA, Houtsmuller AB, Kanaar R, Vermeulen W (2005). "Nuclear dynamics of PCNA in DNA replication and repair". Mol. Cell. Biol. 25 (21): 9350–9359. Shivji KK, Kenny MK, Wood RD (April 1992). "Proliferating cell nuclear antigen is required for DNA excision repair". Cell. 69 (2): 367–74.
- BraA10g007700.3.1C Brassica rapa DNA replication licensing factor MCM2

1 Down-DEG

- BraA02g043920.3.1C Brassica rapa uncharacterized LOC103854884

KEGG ID: ko03440 **Homologous recombination**

3 Up-DEGs

- BraA03g003290.3.1C Brassica rapa replication protein A 70 kDa DNA-binding subunit B (RPA1): binds and stabilizes single-stranded DNA intermediates, that form during DNA replication or upon DNA stress. plays an essential role both in DNA replication and the cellular response to DNA damage. recruits to sites of DNA damage proteins like XPA and XPG that are involved in nucleotide excision repair and is required for this mechanism of DNA repair. RPA plays essential roles in almost all DNA metabolic pathways including DNA replication, transcription, recombination, DNA damage surveillance and recognition, cell-cycle checkpoints, and in all major types of DNA repair including base excision, nucleotide excision, mismatch, and double-strand break repair. Aklilu BB, Culligan KM. Molecular Evolution and Functional Diversification of Replication Protein A1 in Plants. Front Plant Sci. 2016 Jan 29;7:33. doi: 10.3389/fpls.2016.00033.
- BraA09g066020.3.1C Brassica rapa BRCA1-associated RING domain protein 1 (BARD1): E3 ubiquitin-protein ligase. coordinates a diverse range of cellular pathways such as DNA damage repair, ubiquitination and transcriptional regulation to maintain genomic stability.
- Brassica_rapa_newGene_2560

1 Down-DEGs

- BraA02g043920.3.1C Brassica rapa uncharacterized LOC103854884

KEGG ID: ko00480 **Glutathione metabolism**

4 Up-DEGs

http://brassicadb.cn/#/GeneSequence/

- BraA02g038680.3.1C Brassica rapa ribonucleoside-diphosphate reductase small chain C: Catalyzes the biosynthesis of deoxyribonucleotides from the corresponding ribonucleotides. Involved in DNA damage repair and programmed cell death inhibition.
- BraA03g016400.3.1C Brassica rapa glutathione S-transferase F9
- BraA07g027530.3.1C Brassica rapa glutathione S-transferase U23
- BraA09g058780.3.1C Brassica rapa glutathione S-transferase U25

Glutathione S-Transferases (GSTs) in the Biosynthesis of Sulfur-Containing Secondary Metabolites in Brassicaceae Plants (Front. Plant Sci., 13 November 2018 | https://doi.org/10.3389/fpls.2018.01639). GSTs were identified as stress response proteins that accumulated (high inducibility) in response to a wide range of biotic and abiotic stresses since they areca family of Phase II detoxification enzymes that function to protect cellular macromolecules from attack by reactive electrophiles. In addition, conjugation of glutathione (GSH) is required for the Biosynthesis of Glucosinolates.

KEGG ID:ko00240 **Pyrimidine metabolism**
3 Up-DEGs
- BraA01g007870.3.1C Brassica rapa venom phosphodiesterase 2-like
- BraA02g038680.3.1C Brassica rapa ribonucleoside-diphosphate reductase small chain C
- BraA08g018860.3.1C Brassica rapa venom phosphodiesterase 2

KEGG ID: Ko00230 **Purine metabolism**
4 Up-DEGs
- BraA01g007870.3.1C Brassica rapa venom phosphodiesterase 2-like
- BraA02g038680.3.1C Brassica rapa ribonucleoside-diphosphate reductase small chain C
- BraA03g062520.3.1C Brassica rapa adenylyl-sulfate kinase 2, chloroplastic
- BraA08g018860.3.1C Brassica rapa venom phosphodiesterase 2

KEGG ID: ko00270 **Cysteine and methionine metabolism**
4 Up-DEGs
- BraA01g014280.3.1C Brassica rapa cystine lyase CORI3
- BraA03g059780.3.1C Brassica rapa D-3-phosphoglycerate dehydrogenase 1, chloroplastic
- BraA06g008110.3.1C Brassica rapa putative DNA (cytosine-5)-methyltransferase CMT1
- BraA09g055460.3.1C Brassica rapa DNA (cytosine-5)-methyltransferase 1

2 Down-DEGs
- BraA01g036130.3.1C Brassica rapa S-adenosylmethionine synthase 2
- BraA04g017880.3.1C Brassica rapa DNA (cytosine-5)-methyltransferase 1

KEGG ID: ko00591 **Linoleic acid metabolism**
1. UP-DEG

   BraA02g016330.3.1C Brassica rapa lipoxygenase 2, chloroplastic (LOX2): required for synthesis of jasmonic acid (JA) in leave. Bell E., Creelman R.A., Mullet J.E. A chloroplast lipoxygenase is required for wound-induced jasmonic acid accumulation in Arabidopsis. Proc. Natl. Acad. Sci. U.S.A. 92:8675-8679(1995). In plants, the JA signal acts co-operatively with other plant hormones.

KEGG ID: ko00592 **alpha-Linolenic acid metabolism**
1 UP-DEG
- BraA02g016330.3.1C Brassica rapa lipoxygenase 2, chloroplastic (LOX2)

http://brassicadb.cn/#/GeneSequence/

KEGG ID: ko00906 Carotenoid biosynthesis

1 Up-DEG

- BraA09g021450.3.1C Brassica rapa protein LUTEIN DEFICIENT 5, chloroplastic-like (CYP97A3): Heme-containing cytochrome P450 involved in the biosynthesis of xanthophylls. Kim J., Smith J.J., Tian L., Dellapenna D. The evolution and function of carotenoid hydroxylases in Arabidopsis. Plant Cell Physiol. 50:463-479(2009).

KEGG ID: ko00310 **Lysine degradation**

1 Up-DEG

BraA08g018300.3.1C Brassica rapa histone-lysine N-methyltransferase ASHR3

KEGG ID: ko00966 **Glucosinolate biosynthesis**

7 Up-DEGs all are involved in aliphatic GS biosynthesis.

- BraA02g005430.3.1C Brassica rapa 3-isopropylmalate dehydrogenase 1, chloroplastic (IMDH1): (2R,3S)-3-isopropylmalate + NAD^+ = 4-methyl-2-oxopentanoate + CO2 + NADH; Involved in both glucosinolate (methionine chain elongation of glucosinolates) and leucine biosynthesis. He Y., Mawhinney T.P., Preuss M.L., Schroeder A.C., Chen B., Abraham L., Jez J.M., Chen S. A redox-active isopropylmalate dehydrogenase functions in the biosynthesis of glucosinolates and leucine in Arabidopsis. Plant J. 60:679-690(2009)
- BraA02g044250.3.1C Brassica rapa methylthioalkylmalate synthase 1, chloroplastic (MAM1): Determines the side chain length of aliphatic glucosinolate structures. Catalyzes exclusively the condensation reactions of both the first and second methionine carbon chain elongation. Textor S., Bartram S., Kroymann J., Falk K.L., Hick A., Pickett J.A., Gershenzon J. Biosynthesis of methionine-derived glucosinolates in Arabidopsis thaliana: recombinant expression and characterization of methylthioalkylmalate synthase, the condensing enzyme of the chain-elongation cycle. Planta 218:1026-1035(2004).
- BraA03g039430.3.1C Brassica rapa methionine aminotransferase BCAT4 (BCAT4-1)
- BraA03g044200.3.1C Brassica rapa methylthioalkylmalate synthase 1, chloroplastic (MAM1)
- BraA05g028350.3.1C Brassica rapa methionine aminotransferase BCAT4 (BCAT4-2): Converts 2-oxo acids to branched-chain amino acids. Shows activity with L-Leu, L-Ile and L-Val as amino donors and alpha-keto-glutarate as an amino acceptor: 2-oxocarboxylate + L-methionine = 4-methylsulfanyl-2-oxobutanoate + an L-α-amino acid.
  Schuster J., Knill T., Reichelt M., Gershenzon J., Binder S. Branched-chain aminotransferase4 is part of the chain elongation pathway in the biosynthesis of methionine-derived glucosinolates in Arabidopsis. Plant Cell 18:2664-2679(2006)
- BraA06g014390.3.1C Brassica rapa cytosolic sulfotransferase 17 (SOT17): Sulfotransferase that utilizes 3'-phospho-5'-adenylyl sulfate (PAPS) as sulfonate donor to catalyze the sulfate conjugation of desulfo-glucosinolates (dsGSs), the final step in the biosynthesis of the glucosinolate core structure. Piotrowski M., Schemenewitz A., Lopukhina A., Mueller A., Janowitz T., Weiler E.W., Oecking C. Desulfoglucosinolate sulfotransferases from Arabidopsis thaliana catalyze the final step in the biosynthesis of the glucosinolate core structure. J. Biol. Chem. 279:50717-50725(2004)
- BraA07g038970.3.1C Brassica rapa cytosolic sulfotransferase 18 (SOT18)

KEGG ID: ko01210 **2-Oxocarboxylic acid metabolism**

5 Up-DEGs

- BraA02g005430.3.1C Brassica rapa 3-isopropylmalate dehydrogenase 1, chloroplastic (IMDH1)
- BraA02g044250.3.1C Brassica rapa methylthioalkylmalate synthase 1, chloroplastic (MAM1)
- BraA03g039430.3.1C Brassica rapa methionine aminotransferase BCAT4 (BCAT4-1)
- BraA03g044200.3.1C Brassica rapa methylthioalkylmalate synthase 1, chloroplastic (MAM1)
- BraA05g028350.3.1C Brassica rapa methionine aminotransferase BCAT4 (BCAT4-2)

KEGG ID: ko04016 **MAPK signaling pathway – plant**

1 Down-DEG

http://brassicadb.cn/#/GeneSequence/

- BraA03g030100.3.1C Brassica rapa abscisic acid receptor PYL7: a core regulatory component of ABA signaling networks in plants. Di, F., Jian, H., Wang, T., Chen, X., Ding, Y., Du, H., Lu, K., Li, J., & Liu, L. (2018). Genome-Wide Analysis of the PYL Gene Family and Identification of PYL Genes That Respond to Abiotic Stress in Brassica napus. Genes, 9(3), 156. https://doi.org/10.3390/genes9030156.

KEGG ID: ko04075 **Plant hormone signal transduction** [overlap with MAPK signaling pathway – plant]
3 Down-DEGs
- BraA02g043450.3.1C Brassica rapa vegetative storage protein 2
- BraA03g030100.3.1C Brassica rapa abscisic acid receptor PYL7
- BraA03g035760.3.1C Brassica rapa probable inactive chitinase-like protein LaCIC

KEGG ID: ko04626 **Plant-pathogen interaction**
2 Up-DEGs
- BraA03g014590.3.1C Brassica napus ethylene-responsive transcription factor CRF3-like
- BraA03g023590.3.1C Brassica rapa probable WRKY transcription factor 12

KEGG ID: ko03420 **Nucleotide excision repair**
3 Up-DEGs
- BraA03g003290.3.1C Brassica rapa replication protein A 70 kDa DNA-binding subunit B
- BraA07g043590.3.1C Brassica rapa WD repeat-containing protein 76
- BraA09g064370.3.1C Brassica rapa proliferating cell nuclear antigen (PCNA)

1 Down-DEG
- BraA02g043920.3.1C Brassica rapa uncharacterized LOC103854884

KEGG ID: ko03430 **Mismatch repair**
2 Up-DEGs
- BraA03g003290.3.1C Brassica rapa replication protein A 70 kDa DNA-binding subunit B
- BraA09g064370.3.1C Brassica rapa proliferating cell nuclear antigen (PCNA)

1 Down-DEG
- BraA02g043920.3.1C Brassica rapa uncharacterized LOC103854884

KEGG ID: ko03410 **Base excision repair**
1 Up-DEG
- BraA09g064370.3.1C Brassica rapa proliferating cell nuclear antigen (PCNA)

KEGG ID: ko01200 **Carbon metabolism**

2 Up-DEGs

- BraA03g059780.3.1C Brassica rapa D-3-phosphoglycerate dehydrogenase 1, chloroplastic (PGDH1): Involved in the plastidial phosphorylated pathway of serine biosynthesis. Benstein R.M., Ludewig K., Wulfert S., Wittek S., Gigolashvili T., Frerigmann H., Gierth M., Fluegge U.I., Krueger S. Arabidopsis phosphoglycerate dehydrogenase1 of the phosphoserine pathway is essential for development and required for ammonium assimilation and tryptophan biosynthesis. Plant Cell 25:5011-5029(2013)
- BraA06g032840.3.1C Brassica rapa probable 6-phosphogluconolactonase 5 (PGL5): catalyzes the hydrolysis of 6-phosphogluconolactone to 6-phosphogluconate in order to synthesize D-ribulose 5-phosphate from D-glucose 6-phosphate pentose phosphate pathway. Xiong Y., DeFraia C., Williams D., Zhang X., Mou Z. Characterization of Arabidopsis 6-phosphogluconolactonase T-DNA insertion mutants reveals an essential role for the oxidative section of the plastidic pentose phosphate pathway in plant growth and development. Plant Cell Physiol. 50:1277-1291(2009).

http://brassicadb.cn/#/GeneSequence/

KEGG ID: ko00195 **Photosynthesis**

1 Up-DEG

- BraAnng002170.3.1C Brassica rapa uncharacterized ATP synthase C chain-like protein

KEGG ID: ko00500 **Starch and sucrose metabolism**

2 Up-DEGs

- BraA01g007870.3.1C Brassica rapa venom phosphodiesterase 2-like
- BraA08g018860.3.1C Brassica rapa venom phosphodiesterase 2

1 Down-DEG

- BraA08g026680.3.1C Brassica rapa probable alpha,alpha-trehalose-phosphate synthase [UDP-forming] 9

KEGG ID: ko03010 **Ribosome**

2 Up-DEGs

- BraA03g018820.3.1C Brassica rapa venom phosphodiesterase 2
- BraAnng000900.3.1C Brassica rapa genome, scaffold: A03

http://brassicadb.cn/#/GeneSequence/

Table S2. KEGG enrichment analysis of DEGs in γ-PGA relative to Ctrl.

KEGG ID: ko03030 **DNA replication**

10 Up-DEGs same as DEGs in Glu relative to Ctrl.

- BraA02g033700.3.1C
- BraA03g003290.3.1C
- BraA03g042570.3.1C
- BraA03g043620.3.1C
- BraA06g043190.3.1C
- BraA06g044160.3.1C
- BraA07g017290.3.1C
- BraA09g002250.3.1C
- BraA09g064370.3.1C
- BraA10g007700.3.1C

KEGG ID: ko03440 **Homologous recombination**

3 UP-DEGs same as DEGs in Glu relative to Ctrl.

- BraA03g003290.3.1C
- BraA09g066020.3.1C
- Brassica_rapa_newGene_2560

KEGG ID: ko00480 **Glutathione metabolism**

3 UP-DEGs

- BraA02g038680.3.1C Brassica rapa ribonucleoside-diphosphate reductase small chain C
- BraA06g039050.3.1C Brassica rapa ribonucleoside-diphosphate reductase small chain C
- BraA09g005730.3.1C Brassica rapa gamma-glutamylcyclotransferase 2-1

KEGG ID:ko00240 **Pyrimidine metabolism**

3 UP-DEGs

- BraA02g038680.3.1C Brassica rapa ribonucleoside-diphosphate reductase small chain C
- BraA06g020520.3.1C Brassica rapa deoxyuridine 5'-triphosphate nucleotidohydrolase
- BraA06g039050.3.1C Brassica rapa ribonucleoside-diphosphate reductase small chain C

KEGG ID: ko00230 **Purine metabolism**

2 Up-DEGs

- BraA02g038680.3.1C Brassica rapa ribonucleoside-diphosphate reductase small chain C
- BraA06g039050.3.1C Brassica rapa ribonucleoside-diphosphate reductase small chain C

KEGG ID: ko00270 **Cysteine and methionine metabolism**

4 Up-DEGs

- BraA01g014280.3.1C Brassica rapa cystine lyase CORI3
- BraA06g008110.3.1C Brassica rapa putative DNA (cytosine-5)-methyltransferase CMT1
- BraA09g014420.3.1C Brassica rapa methionine gamma-lyase
- BraA09g055460.3.1C Brassica rapa DNA (cytosine-5)-methyltransferase 1

http://brassicadb.cn/#/GeneSequence/

KEGG ID: ko00591 Linoleic acid metabolism

1 UP-DEG

- BraA02g016330.3.1C Brassica rapa lipoxygenase 2, chloroplastic (LOX2)

KEGG ID: ko00592 **alpha-Linolenic acid metabolism**

1 Up-DEG

- BraA02g016330.3.1C Brassica rapa lipoxygenase 2, chloroplastic (LOX2):

1 Down-DEG

- BraA06g043720.3.1C Brassica rapa probable disease resistance protein At5g45510

KEGG ID: ko00906 **Carotenoid biosynthesis**

3 Up-DEG

- BraA01g038240.3.1C Brassica rapa 9-cis-epoxycarotenoid dioxygenase NCED3, chloroplastic: catalyzes the first step of abscisic-acid biosynthesis from carotenoid. Huang et al. 9-cis-Epoxycarotenoid Dioxygenase 3 Regulates Plant Growth and Enhances Multi-Abiotic Stress Tolerance in Rice. Front. Plant Sci., 06 March 2018 | https://doi.org/10.3389/fpls.2018.00162. In the ABA biosynthetic pathway, 9-cis-epoxycarotenoid dioxygenase (NCED) is the key rate-limiting enzyme. Overexpression of OsNCED3 Enhanced NaCl and Water Stress Tolerance and Increased endogenous ABA content.
- BraA05g033460.3.1C Brassica rapa 9-cis-epoxycarotenoid dioxygenase NCED3, chloroplastic
- BraA09g021450.3.1C Brassica rapa protein LUTEIN DEFICIENT 5, chloroplastic-like: Heme-containing cytochrome P450 involved in the biosynthesis of xanthophylls. Kim J., Smith J.J., Tian L., Dellapenna D. The evolution and function of carotenoid hydroxylases in Arabidopsis. Plant Cell Physiol. 50:463-479(2009).

KEGG ID: ko04016 **MAPK signaling pathway – plant**

4 Up-DEGs

BraA02g043450.3.1C Brassica rapa vegetative storage protein 2 (VSP2): vacuolar glycoprotein for temporary storage of nitrogen; accumulation of VSP is induced by ABA and methyJA. Journal of Experimental Botany, Volume 53, Issue 367, 1 February 2002, Pages 265–275, https://doi.org/10.1093/jexbot/53.367.265.

BraA03g035760.3.1C Brassica rapa probable inactive chitinase-like protein LaCIC (CLP): involved in plant resistance against biotic stresses. Ahmed NU, Park JI, Seo MS, Kumar TS, Lee IH, Park BS, Nou IS. Identification and expression analysis of chitinase genes related to biotic stress resistance in Brassica. Mol Biol Rep. 2012 Apr;39(4):3649-57. doi: 10.1007/s11033-011-1139-x.

BraA09g067010.3.1C Brassica rapa pathogenesis-related protein 1A-like: PR genes are significantly induced by both biotic and abiotic stresses, and makes them one of the most promising candidates for developing multiple stress tolerant crop varieties. J.K. Hong, B.K. Hwang. Induction of enhanced disease resistance and oxidative stress Tolerance by over expression of pepper basic PR-1 gene in Arabidopsis. Physiol. Plant, 124 (2005), pp. 267-277. Sajad Ali, Bashir Ahmad Ganai, Azra N Kamili, Ajaz Ali Bhat, Zahoor Ahmad Mir, Javaid Akhter Bhat, Anshika Tyagi, Sheikh Tajamul Islam, Muntazir Mushtaq, Prashant Yadav, Sandhya Rawat, Anita Grover. Pathogenesis-related proteins and peptides as promising tools for engineering plants with multiple stress tolerance. Microbiological Research, 2018, 212–213, 29-37.

Brassica_rapa_newGene_1357

KEGG ID: ko04075 **Plant hormone signal transduction**

1 Up-DEG

- BraA09g067010.3.1C Brassica rapa pathogenesis-related protein 1A-like

3 Down-DEGs

- BraA03g016930.3.1C Brassica rapa integrin-linked protein kinase 1 (ILK1):
- BraA03g030100.3.1C Brassica rapa abscisic acid receptor PYL7: function as abscisic acid sensors. Mediate ABA-dependent regulation of protein phosphatase 2Cs ABI1 (https://onlinelibrary.wiley.com/doi/full/10.1002/advs.202001265). erma Rakesh Kumar, Santosh Kumar Vinjamuri Venkata, Yadav Shashank Kumar, Pushkar Suchitra, Rao Mandali Venkateswara,

http://brassicadb.cn/#/GeneSequence/

Chinnusamy Viswanathan. Overexpression of ABA Receptor PYL10 Gene Confers Drought and Cold Tolerance to Indica Rice. Front. Plant Sci., 2019, 10. doi: 10.3389/fpls.2019.01488 .

- BraA03g037450.3.1C Brassica rapa auxin-responsive protein IAA19: function as repressors of early auxin response genes at low auxin concentrations via interaction with auxin response factors (ARFs), proteins that bind to the auxin-responsive promoter element (AuxRE). Liscum E., Reed J.W. Genetics of Aux/IAA and ARF action in plant growth and development. Plant Mol. Biol. 49:387-400(2002

KEGG ID: ko04626 **Plant-pathogen interaction**

5 Up-DEGs

- BraA02g014980.3.1C Brassica rapa ethylene-responsive transcription factor CRF3 (CRF3): encodes a member of the ERF (ethylene response factor) subfamily B-5 of ERF/AP2 transcription factor family, which is a major group of proteins that are often involved in regulating stress-responses and developmental programs. Component of the cytokinin signaling pathway involved in cotyledons, leaves, and embryos development. Probably acts as a transcriptional activator. Binds to the GCC-box pathogenesis-related promoter element. May be involved in the regulation of gene expression by stress factors and by components of stress signal transduction pathways. Rashotte A.M., Mason M.G., Hutchison C.E., Ferreira F.J., Schaller G.E., Kieber J.J. A subset of Arabidopsis AP2 transcription factors mediates cytokinin responses in concert with a two-component pathway. Proc. Natl. Acad. Sci. U.S.A. 103:11081-11085(2006).
- BraA03g014590.3.1C Brassica napus ethylene-responsive transcription factor CRF3-like: ETHYLENE RESPONSE FACTOR/APETALA2 (ERF/AP2) transcription factors are key regulators of developmental and physiological responses in plants, and they are also often connected with abiotic stress control. Mizoi J., Shinozaki K., Yamaguchi-Shinozaki K. AP2/ERF family transcription factors in plant abiotic stress responses. Biochim. Biophys. Acta. 2012;1819:86–96. doi: 10.1016/j.bbagrm.2011.08.004.
- BraA03g023590.3.1C Brassica rapa probable WRKY transcription factor 12: WRKY proteins are a large family of regulators involved in various developmental and physiological processes, especially in coping with diverse biotic and abiotic stresses. WRKY can regulate multiple sets of genes involved in an interconnected network cooperatively and simultaneously in response to various stimuli. Phu.kan Ujjal J., Jeena Gajendra S., Shukla Rakesh K. WRKY Transcription Factors: Molecular Regulation and Stress Responses in Plants. Front. Plant Sci., 2016, 7. doi: 10.3389/fpls.2016.00760 .
- BraA07g033000.3.1C Brassica rapa calcium-binding protein CML24 (CML24): Ca^{2+} sensor that affects the activity of downstream effectors that synchronize changes in metabolism, gene expression, and turnover of proteins, including transcription factors, protein kinases, metabolic enzymes, and transport protein. La Verde V, Dominici P, Astegno A. Towards Understanding Plant Calcium Signaling through Calmodulin-Like Proteins: A Biochemical and Structural Perspective. International Journal of Molecular Sciences. 2018; 19(5):1331. https://doi.org/10.3390/ijms19051331
- BraA09g067010.3.1C Brassica rapa pathogenesis-related protein 1A-like

4 Down-DEGs

- BraA01g038320.3.1C Brassica rapa protein STRUBBELIG-RECEPTOR FAMILY 7 (SRF7)
- BraA02g034280.3.1C Brassica rapa protein VARIATION IN COMPOUND TRIGGERED ROOT growth response (VICTR): is necessary for inhibition of abscisic acid-induced stomatal closing.
- BraA09g050420.3.1C Brassica rapa PTI1-like tyrosine-protein kinase 3 (PTI13}: represents a new downstream component that integrates diverse lipid and reactive oxygen stress signals and functions closely with a Ser/Thr kinase. Anthony R.G., Khan S., Costa J., Pais M.S., Boegre L. The Arabidopsis protein kinase PTI1-2 is activated by convergent phosphatidic acid and oxidative stress signaling pathways downstream of PDK1 and OXI1. J. Biol. Chem. 281:37536-37546(2006)
- BraA09g065540.3.1C Brassica rapa receptor-like protein 54 (RLP54): consists of an extracellular LRR and transmembrane (TM) domain, as well as receptor-like kinases (RLKs).

KEGG ID: ko03420 **Nucleotide excision repai**r

2 Up-DEGs

- BraA03g003290.3.1C Brassica rapa replication protein A 70 kDa DNA-binding subunit B
- BraA09g064370.3.1C Brassica rapa proliferating cell nuclear antigen

KEGG ID: ko03410 **Base excision repair**

http://brassicadb.cn/#/GeneSequence/

1 Up-DEG

- BraA09g064370.3.1C Brassica rapa proliferating cell nuclear antigen

KEGG ID: ko03430 **Mismatch repair**

2 Up-DEGs

- BraA03g003290.3.1C Brassica rapa replication protein A 70 kDa DNA-binding subunit B
- BraA09g064370.3.1C Brassica rapa proliferating cell nuclear antigen

KEGG ID: ko01200 **Carbon metabolism**

4 Down-DEGs

- BraA05g003040.3.1C Brassica rapa phosphoenolpyruvate carboxylase 2 (PEPC2): oxaloacetate + phosphate = hydrogencarbonate + phosphoenolpyruvate; plays pivotal roles in the carbon fixation of photosynthesis and a variety of metabolic and stress pathways. Cao Jing, Cheng Gang, Wang Lu, Maimaitijiang Tayier, Lan Haiyan. Genome-Wide Identification and Analysis of the Phosphoenolpyruvate Carboxylase Gene Family in Suaeda aralocaspica, an Annual Halophyte With Single-Cellular C4 Anatomy. Front. Plant Sci., 2021, 12. doi: 10.3389/fpls.2021.665279 .
- BraA06g043720.3.1C Brassica rapa probable disease resistance protein At5g45510:
- BraA07g035880.3.1C Brassica rapa pyruvate dehydrogenase E1 component subunit alpha-2, mitochondrial (PDHA2): catalyzes the overall conversion of pyruvate to acetyl-CoA and CO2, and thereby links the glycolytic pathway to the tricarboxylic cycle. Korotchkina L.G., Sidhu S., Patel M.S. Characterization of testis-specific isoenzyme of human pyruvate dehydrogenase. J. Biol. Chem. 281:9688-9696(2006).
- BraAnng000490.3.1C Brassica rapa ribulose bisphosphate carboxylase large chain (rbcL): RuBisCO catalyses the initial step in Calvin's reductive pentose phosphate cycle in plants. Taylor TC, Andersson I. The structure of the complex between rubisco and its natural substrate ribulose 1,5-bisphosphate. J. Mol. Biol. 265, 432-44, (1997).

KEGG ID: ko00195 **Photosynthesis**

1 Down-DEG

- BraA08g035650.3.1C Brassica rapa photosystem II repair protein PSB27-H1, chloroplastic

KEGG ID: ko00500 **Starch and sucrose metabolism**

1 Up-DEG

- BraA10g030330.3.1C Brassica rapa PLASMODESMATA CALLOSE-BINDING PROTEIN 2

2 Down-DEGs

- BraA01g037950.3.1C Brassica rapa probable leucine-rich repeat receptor-like serine/threonine-protein kinase
- BraA08g026680.3.1C Brassica rapa probable alpha,alpha-trehalose-phosphate synthase [UDP-forming] 9

KEGG ID: ko03010 **Ribosome**

5 Up-DEGs

- BraA04g000480.3.1C Brassica rapa genome, scaffold: Brapa_scaffold_48
- BraA06g036740.3.1C Brassica rapa 60S ribosomal protein L5-2
- BraAnng000900.3.1C Brassica rapa genome, scaffold: A03
- BraAnng001460.3.1C Brassica rapa genome, scaffold: Brapa_scaffold_54
- BraAnng001470.3.1C Brassica rapa subsp. nipposinica mitochondrial DNA, complete sequence, cultivar: Oushou hakusai

1 Down-DEG

http://brassicadb.cn/#/GeneSequence/

- BraA08g032990.3.1C Brassica rapa 60S ribosomal protein L21-1
